# Contribution of the infection ecosystem and biogeography to antibiotic failure in vivo

**DOI:** 10.1038/s44259-024-00063-2

**Published:** 2024-12-04

**Authors:** Rahan Rudland Nazeer, Isabel Askenasy, Jemima E. V. Swain, Martin Welch

**Affiliations:** Department of Biochemistry, University of Cambridge, Cambridge, UK

**Keywords:** Target identification, Drug development, Antibiotics

## Abstract

The acquisition of antibiotic resistance in bacteria, though a deeply concerning international issue, is reasonably well-understood at a mechanistic level. Less well-understood is why bacteria that are sensitive in vitro to well-established and widely-used antibiotics sometimes fail to respond to these agents in vivo. This is a particularly common problem in chronic, polymicrobial infection scenarios. Here, we discuss this in vitro-in vivo disconnect from the perspective of the bacterium, focusing in particular on how infection micro/macro-environment, biogeography, and the presence of co-habiting species affect the response to antibiotics. Using selected exemplars, we also consider interventions that might improve treatment outcomes, as well as ecologically ‘eubiotic’ approaches that have less of an impact on the patient’s commensal microflora. In our view, the accrued data strongly suggest that we need a more comprehensive understanding of the in situ microbiology at infection sites.

## Introduction: the problem

Antibiotics have contributed enormously to the improvements in life expectancy during the 20^th^ Century and remain the primary “go to” option for treating bacterial infections. However, on the horizon, a crisis is approaching: the rise of antibiotic resistance, responsible for over one million deaths in 2019 and more to come^1^. There has been a great deal of movement within universities and SMEs—although conspicuously less by larger pharmaceutical enterprises^2^—to understand and implement solutions to this impending crisis. These solutions range from fortification of the immune system to better combat microbial invasion^3^ through to altering the pharmacokinetics and toxicity of existing or new antimicrobial agents^4^. Furthermore, antibiotics that were previously considered as peripheral to the mainstream (such as antimicrobial peptides) are now being re-evaluated and their arsenal expanded^5^, and new chemistries are being developed to deliver polymer nanobiotics^6^ or even ‘microrobots’^7^. For an up-to-date discussion of some of these alternatives to ‘traditional’ antibiotics, see the recent insightful review by MacNair et al.^8^. It is also worth pointing out at this juncture that antibiotic candidates *are* making it through to clinical trials: the problem is that the attrition rate (often between trial phases 1 and 2) is very high. This leak in the pipeline is almost always due to safety/toxicity issues^1^. Nevertheless, and in spite of these advances, our understanding of pathogen behaviour *at the infection site* remains limited, and proven, on-the-market antibiotics that work well in vitro can sometimes fail in vivo for reasons other than host toxicity. Here, we assess the possible reasons for this, with a particular focus on the contribution of the infection site micro- and macro-environment and ecology.

For clarity, in this commentary, we do not aim to discuss why drugs in the development/testing pipeline fail to reach the clinic. Nor do we directly address pharmacokinetic/pharmacodynamic (PK/PD) issues (although these are touched upon) or antibiotic exclusion from certain tissues (e.g., the CNS or prostate gland). Rather, we aim to discuss why well-established, on-the-shelf existing antibiotics sometimes fail to resolve infections in patients, even when they are applied in doses that should be lethal to the infecting organism. By way of example, this is typified by chronic infections. In recent years, such recalcitrant clearance has often been attributed to biofilm formation, although as we point out below, while biofilms do play a role, they are not the sole arbiters of the problem.

Our primary focus is not on resistance per se, but on antibiotic ‘tolerance’^9–11^ (sometimes also described as ‘resilience’^12^). Here, a population of nominally susceptible bacteria manage to survive challenge with would-be lethal concentrations of antibiotics. Tolerance is distinct from persistence and resistance. Persistence is associated with mechanisms that permit survival of a *sub-population* of cells in response to antibiotic challenge, whereas resistance is associated with the acquisition of genes/mutations that enable survival of the entire population in the presence of high levels of the antibiotic. By contrast, tolerance is a non-heritable phenotypic phenomenon (although some mutations can pre-dispose towards increased tolerance) that has been widely observed in both laboratory and clinical environments^13,14^. Intriguingly, tolerance is sometimes associated with the subsequent development of full-blown resistance^15–17^. The molecular basis for tolerance remains unclear, although it is likely to be multifactorial and is contingent on the circumstances that the bacteria find in themselves.

**Fig. 1:**
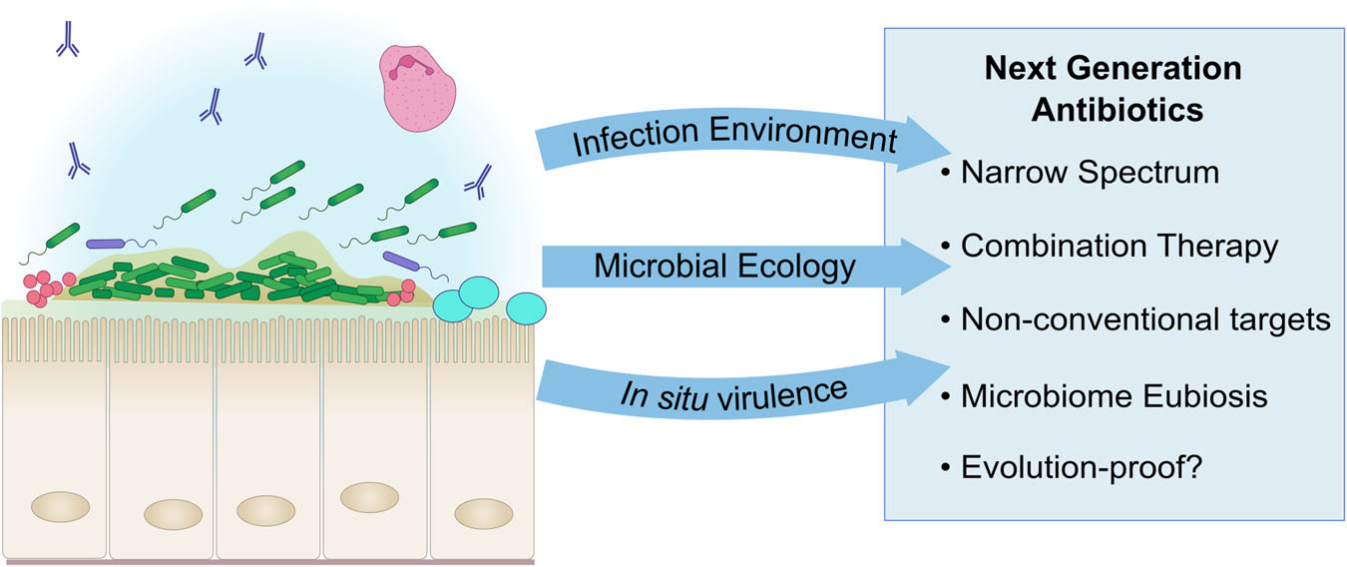
How the “in vivo bacterial experience” at the infection site might be better exploited in the development of improved antibiotic interventions. The figure summarises how future generations of antibiotics might be developed to take into account the issues discussed in this perspective, with a particular focus on the selection pressures at the infection site, co-habiting microbes, and protection of the commensal microbiota.

To help us address this issue, we ask (paraphrasing Thomas Nagel^18^), ‘what is it like to be a microbe during an infection?’ This is a complex question to address, encompassing factors relating to both the host and the microbe. For simplicity, we therefore focus on just three key issues concerning *microbial* physiology and ecology during an infection (summarised in Fig. 1).

- Why do nominally susceptible bacterial species sometimes respond differently to antibiotics in infection scenarios compared with their response in vitro?
- How can we improve antibiotic efficacy in infection scenarios?
- Are there any unforeseen consequences of antibiotic usage and can we exploit ecological and evolutionary thinking to circumvent these?

## Why bacterial infections sometimes respond poorly to antibiotic treatment: influence of the infection site micro- and macro-environment

A holistic view of infection biology should take into account environmental factors, the impact of co-habiting microbes, and the host immune response to the infection, among others. [Indeed, oftentimes, it is the immune cells that do much of the *de facto* bacterial clearance from infection sites; antibiotics just facilitate this by diminishing the bacterial load]. However, another major factor that underpins antibiotic failure relates to the ‘obstinacy’ of the bacterium itself. This obstinacy is composite—partly accounted for by the growing reservoir of antibiotic resistance determinants circulating in the global gene pool (beyond the scope of the current discussion), and partly by the bacteria’s own phenotypic tolerance to antimicrobial challenge. But might the biogeography at an infection site itself also influence tolerance?

### Host biogeography

The chemical and spatial heterogeneity associated with infection sites can lead to corresponding heterogeneity in the population of infecting bacterial cells^19^. For example, the airways of people with cystic fibrosis (CF) are often chronically infected with *Pseudomonas aeruginosa*. However, in any given infected patient, different lobes of the lung appear to support distinct sub-populations of the organism, with limited mixing between populations observed. Further complicating the situation, the *P. aeruginosa* lobe-specific adaptations in one patient are not necessarily seen in the same lung lobe in other patients. Of relevance to the current discussion, many of these patient-/lobe-specific adaptations appear to affect genes known to be involved in antibiotic resistance, the response to host defences, and virulence^19^. Moreover, and even when a pathogen can be successfully cleared from the primary site of infection, re-infection can still occur *via* seeding from other tissues in the host^20^. For example, *P. aeruginosa* can colonise the nasopharynx and from there migrate into the lungs, resulting in more serious infection^21^. In addition, it is worth recalling that antibiotic efficacy differs at different sites within the body, often due to varied levels of tissue penetration—although there remains a lack of robust data that specifically defines these differences^22^.

### Biofilm structure necessarily introduces physiological heterogeneity

Another contributory factor towards infection heterogeneity is biofilm formation, which is often associated with chronic infections. For example, in the chronically-infected CF airways just mentioned, microbial biofilms manifest as free-floating clumps (usually comprising just a few hundred or a few thousand cells) suspended in the airway mucus. By contrast, in other infection scenarios they form quasi-two-dimensional coatings on tissues or surgical implants^1,23^. Irrespective of their morphology, biofilm structures are associated with steep, self-generated gradients of oxygen. These gradients have wide-ranging effects on metabolism, growth rate, and antibiotic uptake^24^. [Since most antibiotics target macromolecular biosynthesis, they are generally more effective on rapidly-growing cells than on slow-growing cells. Furthermore, some antibiotics, such as aminoglycosides, require a proton-motive force for uptake—again, meaning that metabolically more active cells are more susceptible^25^]. This heterogeneity also extends to the matrix of the biofilm. The three main matrix exopolysaccharides found in *P. aeruginosa* biofilms are known to be differentially immunogenic^26^, and their heterogeneous distribution across the structure may play a role in minimising immune responsiveness at the biofilm periphery, while simultaneously preserving the internal matrix characteristics conferred by other, more inflammatory exopolymers^27^.

### Shared goods in the biofilm hinterland

The conventionally strict intracellular-extracellular biochemical divide is not always respected in biofilm aggregates either, since the biofilm matrix represents a “gray zone” that lies between the true extracellular milieu and the region immediately surrounding the cells. Perhaps a better way of thinking about this is to consider how goods can be “locally shared” in multicellular communities. A good exemplar is the phenazine-mediated extracellular electron transport utilized by many redox-active microbial species, which facilitates the state of metabolic heterogeneity described above, and consequently, antibiotic tolerance^28^. Crucially, the expression of such antibiotic sensitivity-conferring factors is not only uneven within sessile bacterial communities; it is also often exquisitely responsive to local environmental stimuli. The upshot of all this is that, in many infection scenarios involving biofilms, we would fully expect a high degree of variation in responsiveness to antibiotics.

### Intrinsic cellular heterogeneity

There is, however, much more to the problem than this. Using single-cell approaches, we can superficially strip away the confounding factors associated with self-generated (or exogenously imposed) environmental variation, allowing us to examine intrinsic variations in the antibiotic responsiveness of individual cells^29^. Such approaches have revealed that genetically identical cells can exhibit a wide range of susceptibilities to common antibiotics^30^. These differences have been linked with stochastic variations in gene expression, and unequal partitioning of cytoplasmic matter during division^31–35^; problems that can now be experimentally accessed through single cell ‘omics^36^.

### Infection sites themselves are inherently “resistagenic”

Microbial cells stressed by the presence of sub-lethal concentrations of antibiotics, and the host immune cells themselves, both produce copious quantities of reactive oxygen species (ROS). Although these ROS are primarily intended to be cytotoxic, like any free radical, they also damage microbial DNA (hence accelerating the vertical acquisition of resistance). Not surprisingly, efforts are afoot to reduce the rate of acquisition of resistance by suppressing drug-induced mutability^37^. More recently, ROS have been shown to stimulate the exchange of DNA between microbes (promoting horizontal dissemination of resistance among the population)^38^. Such horizontal gene transfer (HGT) need not necessarily be limited to just a single species: harmless commensals are now known to act as reservoirs of resistance genes^39^, although relatively little is understood about how they transfer these genes to co-habiting pathogens. In this regard, there is a desperate need for experimentally accessible/perturbable models of polymicrobial infections to study such phenomena. Somewhat counterintuitively, the metric of success for such models may be their capacity to capture failure; in vitro models that faithfully recapitulate the antibiotic failures witnessed in clinics^40^ may prove to be more trustworthy^40^.

### In vitro growth media rarely capture in vivo growth conditions

Aside from the structural heterogeneity associated with infection sites, another contributor to the in vitro-in vivo disconnect is the selection of growth media used to test antibiotic activity. Although widely-available rich media such as Brain-Heart Infusion (BHI) broth provide a convenient reference “benchmark” for antibiotic activity, such media do not accurately capture the chemical environment at many infection sites. Clearly, a better solution would be to employ media that more faithfully recapitulate the host environment^41^. For example, why use a medium derived from hydrolysed cow organs when trying to test an antibiotic destined to treat the lung? In those circumstances, surely artificial sputum medium would be more physiologically appropriate? Similarly, artificial urine medium would be a better option for agents targeting urinary tract infections, and chronic wound medium for soft tissue infections, and so on.

## Can we improve antibiotic outcomes in infection scenarios? What should we target?

In this section, we ask whether there are ways in which antibiotics might be made more efficacious in infection scenarios? A popular approach to this has been to target the mechanism(s) associated with antibiotic resistance e.g., by inhibiting efflux pumps, β-lactamase activity, and so on. However, and as outlined earlier, in the current commentary we are not concerned with the phenomenon of resistance per se. Also progress with so-called “resistance breakers” has been extensively reviewed elsewhere^42^, so for the remainder of this section we focus on alternative approaches for adjuvant therapy, drawing exemplars from the field of bacterial virulence. [In medicine, adjuvants are agents that increase the efficacy of other compounds].

### Stress mitigation

One medium-term proposition has been to restrict the mutagenic effects of conventional antibiotic treatments to their intended targets, the bacteria—this topic has been recently reviewed by Batchelder and colleagues^43^. Some such suggested approaches include the administration of adjuvants alongside antibiotics (to simultaneously sequester and quench ROS, for example) or the cellular responses that such stressors elicit^43^. In this regard, we note that the worst-offending ROS (hydroxyl radicals) react with just about any biomolecule, which raises questions about the likely efficacy of such approaches. Nevertheless, widespread employment of stress-mitigation adjuvants *may* provide a temporal window for the research community more time to find more effective long-term solutions.

### Targeting virulence

Another approach is to target those aspects of bacterial physiology that are directly associated with disease itself, such as virulence. Anti-virulence therapies certainly hold promise, although there are a priori concerns that have yet to be resolved: ‘virulence’ is tricky to adequately define, and the expression of some virulence factors is context-dependent (and consequently, are often subject to very specific selection pressures^44^). Moreover, and in spite of the best hopes of the practitioners of genomics, virulence remains difficult to predict based on genetic inventory alone^45^. Also, the pathogenicity of some organisms is often determined by multiple virulence factors, so targeting these one at a time is unlikely to have a major impact. [This notwithstanding, in some cases inhibition of single virulence determinants has shown promise. For example, for some pathogens, Type III secretion is the “nuclear button” in their arsenal, making this an obvious target for drug development^46,47^]. Finally, virulence factors, and especially their regulatory pathways are often organism-specific, so drugs that are efficacious for one species do not necessarily show activity on others. Nevertheless, such efforts may still be worthwhile for the more challenging ESKAPE pathogens.

Even when virulence factors can be identified and successfully inhibited in vivo, the outcomes are not always beneficial for the host. For example, it should be borne in mind that planktonic cells generally secrete far more virulence factors than their sessile, biofilm-associated cousins. Consequently, biofilm disruptors can potentially have unanticipated adverse consequences. This was vividly illustrated in a mouse wound infection model following treatment with a biofilm matrix disruptor, which led to lethal septicaemia rather than immune clearance^48^.

### Targeting quorum sensing

Over the last couple of decades, the master regulators of virulence have become a popular target for antibacterial development. A case in point is quorum sensing (QS), a cell-cell communication mechanism mediated by small diffusible molecules (autoinducers). In some pathogens such as *P. aeruginosa* or *Serratia marcescens*, activation of QS signalling pathways leads to the expression of multiple virulence factors, facilitating infection, persistence, and evasion of the host’s immune response^49^. Although rather few pathogens are known to employ QS to regulate virulence, those that do are often a major clinical problem, and the targeting of QS in these species nicely highlights both the advantages and the limitations of such adjuvant interventions.

QS enables the concerted expression of tissue-degrading virulence factors (and their associated secretion machineries), proteinaceous toxins, and secondary metabolites. It also promotes up-regulation of certain multi-drug efflux-pumps, and, under some conditions, biofilm formation^50,51^. As noted above, inhibition of the latter is potentially counter-productive, since the released planktonic cells are more virulent than their biofilm-associated counterparts. However, the dual effect of QS inhibitors in suppressing virulence as well as biofilm formation should, in principle, take care of this problem. Furthermore, it is envisaged that any increased titre of antibiotic-susceptible planktonic cells might be dealt with through co-administered conventional antibiotics^52,53^. Interestingly, there is also evidence from animal models that direct inhibition of QS can aid immune clearance of infections^54,55^, adding another layer of potential utility to such adjuvant interventions. Proposed modes of QS inhibition include interfering with the biosynthesis of autoinducers, their stability, or their ability to bind to the cognate receptor molecule (usually through competition)^56–58^. More recently, researchers have also developed approaches that empty the cells of the signal molecules they have accumulated^59^.

### Anti-QS adjuvants are not “evolution proof”

An argument that could (and has been) made is that, since QS is not essential to the cell, the selection pressure leading to resistance will be lower than for conventional antibiotics. This has led to the suggestion that anti-QS interventions might be more ‘evolution proof’ than “conventional” antibiotics, especially when the two interventions are used in combination^44^. However, we note that the secretion of metabolically-costly QS signals—in the hope that they will accumulate in the culture and trigger a similarly resource-intensive secretion of exo-enzymes—is, in itself, a significant metabolic gamble. [All the more so, given that the cell has no control over whether these secreted enzymes will encounter some nearby host tissue to degrade]. We further note that all this occurs as the cells enter stationary phase i.e., just at the point where resources become limiting in the culture. In this regard, QS-dependent virulence factor secretion is simply a means-to-an-end to secure more nutrients when these become limiting, but it remains a gamble, nonetheless. Mitigating that, the fact that this mechanism has been selected for in evolution is evidence that the strategy works, although it is certainly not “cost-free”. Indeed, and commensurate with this, QS-deficient mutants are known to arise with high frequency at some infection sites. For example, *P. aeruginosa* mutants deficient in the central QS regulator, *lasR*, are a common feature in the airways of people with CF^60^, and this has been linked with worse patient outcomes^61^. It is currently difficult to entirely explain this observation, although some researchers have suggested that *lasR* mutants may be ‘social cheats’; mutants that should inevitably arise when “public goods” such as secreted virulence factors are being produced^62,63^. In vitro, such cheats have a fitness advantage, as they are not burdened by the resource drain associated with up-regulated expression of virulence factors; yet, at the same time, can sup off the investments made by QS-proficient cells in the culture. However, it is also worth noting that, in vitro, virulence is readily restored in *lasR* mutants through the acquisition of secondary mutations (e.g., in *mexT*)^64,65^. QS inhibition is therefore not necessarily an “evolution proof” strategy, although currently, not enough is known about the fitness costs associated with such “QS bypass” mechanisms.

Another complication in QS inhibition is its redundancy, especially in pathogens such as *P. aeruginosa*, which deploy a hierarchical array of autoinducers each with overlapping regulons^66^. In other bacteria, such as *Vibrio* spp., QS is characterized by a parallel network of receptors that titrate different signals and enable exquisite fine-tuning to different environmental stimuli^50^. Similarly, in the case of *Streptococcus pyogenes*, the QS response is mediated by a pair of antagonistic signalling systems^50^. The take-home message is that there is no one-size-fits-all anti-QS strategy^57,66^.

### Phage therapy

The dearth of antibiotics in the pipeline has also led to a resurgence of interest in bacteriophage therapy as an alternative^67^. Despite nascent success in the early 20^th^ Century, particularly in the Soviet Union, phage therapy was largely overlooked by the Western scientific community following the advent of penicillin^67^, and even now, they are often seen as a “last resort” option. [Currently, the main obstacle to their more widespread deployment seems to be a largely legislative one, although this may be changing as the need for alternative antibacterial therapies becomes more acute.] As natural predators of bacteria, phage have evolved to invade their hosts, replicate, and then exit *via* cell lysis. Crucially, although phage resistance can easily arise e.g., due to mutation of the receptor molecules on the bacterial cell surface, as the most abundant biological entities on the planet they represent a huge and relatively untapped resource for biopanning. Moreover, and in an effort to circumvent the problem of resistance, when phage are used as therapeutics they are often supplied as a “cocktail” in much the same way that two or more antibiotics are sometimes given in combination: here, resistance to one agent is abrogated by the presence of a second. Further mitigating the problem, as noted by Oromí-Bosch et al., phage engineering can be applied to minimize resistance^68^.

Phages are potentially advantageous in that their narrow host range means that they are minimally disruptive to the commensal microbiome (an issue that we discuss further below). However, phage therapies, in spite of being superficially more “evolution proof” than anti-QS therapies, are not without shortcomings. Many phage are active only on very specific strains of a given species, so detailed typing is required. Storage and formulation are a problem too: these are not “off-the-shelf” solutions, although they do offer promise in the personalized medicine sphere. Furthermore, the immune response that inevitably accompanies introduction of a given phage cocktail *potentially* precludes the use of the same cocktail, even if it is effective, in future treatments^68^.

## Ecological and evolutionary thinking—long term strategies

Due to their remarkable evolutionary and phenotypic plasticity, microbes are “moving targets” for therapeutic intervention^69^, so the evolutionary dynamics of an in vivo infecting population are often complex.

### Influence of co-infecting strains

In addition to the intrinsic diversification linked with ongoing mutation/selection, susceptible hosts can sometimes get co-infected by multiple strains of a given pathogen. For some species such as *Mycobacterium tuberculosis*^70^, co-existence between these variants may occur transiently, but in the long-term, the population is relatively homogenous. By contrast, in the case of *P. aeruginosa*, co-existence among different strains is relatively well documented, and in these circumstances, the emergence of antibiotic resistance is facilitated^71^. Although we still understand relatively little about this long term adaptation, recent developments are beginning to shed light on the evolutionary dynamics at infection sites.

### Influence of co-infecting species

In addition to intra-species variation, another important factor for consideration is the presence of co-infecting species—and of the commensal microbiome living alongside the focal pathogen. Such polymicrobial infections are common, and are often a hallmark of chronic, long-term infections, yet they remain poorly understood^72^. The vast majority of microbiology research since inception of the discipline has focussed on single species in axenic culture^73–76^, and as such, our understanding of antimicrobial efficacy in the context of a polymicrobial milieu is distinctly lacking^77,78^. For an in-depth examination of the state of this field, see the recent review by Bényei et al.^79^.

Inter-species interactions can increase resistance or promote tolerance in a myriad of ways^40,72^. For example, exotoxins secreted by *P. aeruginosa* select for small-colony variants (SCVs) of *Staphylococcus aureus*. These SCVs are known to be resistant to aminoglycosides^80^, such that co-habiting *P. aeruginosa* has the potential to decrease the efficacy of aminoglycosides against *S. aureus*. Conversely, co-habiting species can sometimes also have the opposite effect, increasing susceptibility to antibiotics^72^, and we are only just beginning to uncover how antibiotics affect the ecology of more complex polyspecies communities. Fortunately, improved experimental models^81^, and better descriptive and predictive models of infection ecology^82^ look set to change this situation.

### Improved animal models

The use of animal models for the study of pathogenic microbes has been reviewed elsewhere^83,84^, so we will not discuss their advantages and limitations here. However, it is worth noting that not all such models faithfully capture the disease dynamics associated with human hosts. Recent years have seen progress in this respect, with attempts to ‘humanise’ animal models (mainly mice) in various ways. For example, the gastrointestinal tract of germ-free humanised mice can now be colonised using human faecal samples, resulting in a microbiome that more closely reflects the human gut flora^85,86^. Such mice have been used by Collins and colleagues to investigate susceptibility to *Clostridioides difficile* infection following antibiotic treatment^87^. In parallel, Lewin et al. have developed a framework that assesses model accuracy by comparing pathogen gene expression in the model to that in the equivalent human infection environment^88^. This iterative approach has also been used to alter a well-established mouse acute pneumonia model, enabling it to better capture features of chronic *P. aeruginosa* lung infection in CF^89^.

### Polymicrobial infections are complex

By definition, polymicrobial infections involve more than one species. However, chronic infections are also often associated with diversification of the infecting microbes, so a proper understanding needs to consider both intra- and inter-species interactions. To further complicate things, often, the basic biology of many of these species is only poorly understood. It is only in recent years (with the routine use of culture-independent approaches such as 16S rDNA analyses) that it is becoming clear that certain species are consistently present at some infection sites (e.g., the anaerobes in infected CF airways). These species are often understudied and hard to cultivate, so we know very little about their biology^90,91^. There is also an increasing appreciation that certain species may act as “keystones”; organisms that are present at low titres, but which have a disproportionate impact on the dynamics of the whole ecosystem. Clearly, a better understanding of these “influencers” may offer a line-of-sight towards improved patient outcomes.

We also need to be cognisant of the effect that antibiotics have on inter-species dynamics in polymicrobial infections. For example, in some circumstances, it turns out that maintenance of a diverse polymicrobial load is associated with a better prognosis for the patient^92^. Over time, polymicrobial diversity declines, and the pathogen(s) present begin to dominate. The extent to which long-term antibiotic usage contributes towards this decline in diversity is not clear, although antibiotics are known to have a substantial (usually, negative) impact on commensal species. Antibiotics that specifically target the pathogen(s) present, and not the commensals, may help to maintain a healthy eubiotic ecosystem (eubiosis describes a balanced microbial community; dysbiosis an unbalanced one). This is important because antibiotic-induced dysbiosis can itself lead to disease—an archetypal example being the bloom of *C. difficile* following decimation of the gut microflora by oral antibiotic treatment. Somewhat counter-intuitively, the antibiotic treatment here results in more severe diarrhoeal disease.

### Smart antibiotics

In an effort to minimize dysbiosis, Muñoz et al.^93^ constrained their search for new drugs to those that target a lipoprotein transporter found in Gram-negative bacterial species. Crucially, the amino acid sequence of this transporter is conserved in many pathogens but not in commensal species. Beyond demonstrating efficacy of the drug against the target pathogens, they also tested it against an array of commensal organisms—not only in vitro, but also in a mouse-microbiome model—and determined that their drug’s efficacy did not come at a cost to the Shannon diversity of the gut microbiome. The authors also pointed out additional examples of where this tactic might be further deployed.

### Biological interventions

Long-term antimicrobial approaches need to be both ecologically and evolutionarily mindful. Mutlu et al.^94^ provide a neat contemporary example of the former by showing that antibiotic-sensitive QS mutants of *P. aeruginosa* can be introduced into a murine chronic wound infection scenario populated by antibiotic-resistant cells. As the QS mutants invade the existing population of resistant cells, titres of the latter decrease, leading to progressive sensitization of an otherwise resistant population. They further showed that co-administration of conventional antibiotics alongside these presumed “social cheaters” led to improved resolution of the infection. Although this is a useful proof-of-principle outcome, we remind the reader that there is likely more to the story that this, since QS mutants arise with high frequency anyway in a cadre of people who are aggressively treated with antibiotics (people with CF), so it remains to be seen whether the general principle has any therapeutic benefit in other infection contexts. Also, legislation addressing biological interventions is lagging far behind that of chemical interventions, although progress made in implementing phage therapy is likely to accelerate change on this front.

## Conclusion

Most antibiotics work well and clear infections most of the time. However, sometimes, they fail to live up to expectation, even when there is no obvious reason for this. As discussed in the current commentary, a major contributor to this is likely the characteristics of the infection site itself. Indeed, we currently understand remarkably little about the in situ biology of infections, and there is also an urgent need to develop improved experimental and in silico models that faithfully capture infection site microbial dynamics (inclusive of intra- as well as inter-species dynamics). However, with advances being made in adjuvant therapy and in the development of potent antibiotics that preserve commensal microfloral communities, we remain buoyant about prospects for the future.
